# Adherence to supportive periodontal treatment in relation to patient awareness

**DOI:** 10.4317/jced.59035

**Published:** 2022-01-01

**Authors:** María Navarro-Pardo, Cecilia-Fabiana Márquez-Arrico, Alba Pallarés-Serrano, Francisco-Javier Silvestre

**Affiliations:** 1 Private dentist, postgraduated student University Hospital Doctor Peset, 46017 Valencia, Spain; 2 Department of Stomatology, University Hospital Doctor Peset-FISABIO, 46017 Valencia, Spain; 3 Department of Periodontics, Catholic University of Valencia, 46001 Valencia, Spain; 4 Department of Stomatology, University of Valencia 46010 Valencia, Spain

## Abstract

**Background:**

To evaluate the risk profile of noncompliant patients in relation to adherence to supportive periodontal therapy in order to identify factors associated with this profile, and be able to prevent the abandonment of periodontal therapy.

**Material and Methods:**

This cross-sectional observational and comparative study was carried out on the patients who attended the Periodontics department of a University in Valencia (by a questionnaire and followed-up the periodontal supportive therapy through the medical history.) 220 patients were interviewed and gave their informed consent and data release permission before taking part in the study, which was approved by the Ethics Committee (UCV/2019-2020/048).

**Results:**

48.84% of self-reported patients were regular compliers, in contrast with 10.62% of referred patients. Those with acute symptoms were greater adherent than those patients who didn´t present symptoms. Regarding patients undergoing surgical procedures, significant results were obtained: 69.70% showed adherence, in contrast with 18.67% patients with basic treatment. Results between men and women were similar. However, the age of the non-compliant patients was slightly older.

**Conclusions:**

Self-reported patients presented a significantly higher degree of adherence to periodontal supportive therapy than the referred patients. Patients with acute symptoms presented higher adherence than those without them. Patients who underwent surgery presented a significantly higher degree of adherence than patients who received basic periodontal treatment. No conclusive data have been found regarding sex and age.

** Key words:**Awareness, periodontal disease, compliance, SPT.

## Introduction

Periodontal diseases can be defined as inflammatory disorders caused by bacteria that affect the periodontium, which is composed of the gum, periodontal ligament, cementum and alveolar bone. There are two main types of periodontal diseases: gingivitis, a mild and reversible form, and periodontitis, when inflammation progresses deeper into the tissues with the formation of periodontal pockets and irreversible destruction of the periodontal ligament and bone damage which, in advanced cases, can lead to tooth loss (1,2).

Periodontal disease (PD) is considered a public health problem due to its high prevalence (it affects more than 50% of the adult population, making it the sixth most prevalent disease in the world) (3), its impact on quality of life (related to self-esteem and well-being) and the high cost of the treatment (4).

Periodontal treatment It includes behavioral change techniques, such as: oral-hygiene instructions; smoking-cessation and dietary intervention (5,6). These, followed by nonsurgical periodontal treatment (subgingival instrumentation to remove plaque and calculus) has been shown to control periodontal infection and to arrest progression of the disease in a significant number of cases. However, despite completion of nonsurgical treatment, a number of periodontal pockets, often remain. Therefore, surgical treatment is needed (7). Finally, the ongoing Supportive Periodontal Treatment (SPT), which begins once the active treatment just mentioned is completed. It involves regularly scheduled sessions depending on the long-term effectiveness of the treatment due to the chronic and multifactorial nature of the disease (5,6,8-11).

A problem facing periodontal treatment is the low adherence undergoing SPT once patients have completed the active part. Moreover, PD has been seen to be difficult to diagnose by patients making them not even start the treatment. The ability of patients to determine if they have PD known as ‘’self-reported’’ patients is crucial, as recognition of the symptoms and/or signs of PD enables patients to seek help and treatment (12). Warning signs of PD can be gingival recession, mobility, bleeding gums or symptoms such as halitosis, sensitivity and pain. However, specialist treatment is not always sought due to the belief that tooth loss and mobility are inextricably linked to ageing (13-15). Most common reason for seeking medical and dental treatment are pain and discomfort like dental cavities, PD rarely causes acute pain, therefore, patient motivation towards periodontal treatment can be a challenging aspect (16).

Once a patient has been diagnosed with PD, great attention has been paid to identify the variables that affect treatment adherence like behavioral, cultural and economic factors. In addition, factors such as age, gender, type of treatment and patient satisfaction have been seen that could affect patient behavior (17-19).

Adherence is defined by the World Health Organization (WHO) as “the extent to which the patient follows medical instructions” (20) and includes the implication and commitment by the patient with his/her disease, its treatment and the therapist (15). To improve adherence patients must acquire an active role in the management process of the disease, increase their autonomy and their capacity for self-care. They need to know the disease and understand it, as well as the prescribed treatment and the importance of fulfilling it. Despite the fact that prevention and treatment of this type of disease is nowadays predicTable and successful, recent epidemiological studies have indicated a high lack of knowledge on the part of the population about PD (13,15,21).

As it has been found, periodontal awareness influences daily oral hygiene practices and routine periodontal care (22). Because of this, in the following investigation we studied the association between adherence to the SPT and the patients profile.

## Material and Methods

This prospective observational and comparative study was carried out following the Strengthening the Reporting of Observational studies in Epidemiology (STROBE) criteria on a total of 196 patients who were diagnosed with PD in the clinics of the Catholic University of Valencia from September 2016 to April 2019. Treatment follow-up was assessed by the clinical history in addition to a questionnaire to the patient about the reason for consultation and knowledge about PD. Participants gave their informed consent and data release permission before taking part in the study, which was approved by the Ethics Committee: UCV/2019-2020/048 where a favorable report was agreed.

Inclusion criteria were patients over 18 years of age, who had received basic active periodontal treatment (R.A.R) or surgery, from September 2016 to April 2020 and had been undergoing SPT for at least one year. Exclusion criteria were having any type of mobility impairment due to lack of dependability, terminally ill patients, pregnant women and patients with diseases that could affect the immune system such us HIV.

- Adherence to the SPT 

The main variable to be examined was the degree of adherence to the SPT; this information was obtained by accessing to each patient’s clinical history. The patients were classified according to the pattern of adherence to the SPT appointments: (23). Regular compliers (RC) if they were 100% compliant with the visits. Erratic compliers (EC): if they had not attended 50% of the scheduled SPT visits but had continued irregularly, and non-compliers (NC): patients who dropped out of the SPT.

- Reason for consultation 

The reason for consultation was obtained by means of a validated patient questionnaire in which all the information was completed and the patients were classified into two groups: self-reported: (24) patients who attended on their own, concerned about the presence of some sign or symptom that they considered to be “not normal”, characteristic of PD such as bleeding on brushing or spontaneous bleeding, visual signs of inflammation, gingival retraction or pockets, mobility of some dental piece, sensitivity, bad taste in the mouth, halitosis, pain or discomfort. The other group was patients referred for a reason other than periodontal disease (24).

- Secondary variables 

Other variables that were evaluated were the absence or presence of pain, where patients were classified as having acute pain or being asymptomatic (15). They were also classified according to whether they had received nonsurgical or surgical treatment (23) as well as the sex (25) and mean age of each group of patients (1).

- Sample size calculation

It was calculated that a random sample of 206 individuals would be sufficient to estimate, with a 95% confidence interval (CI) and a precision of +/- 0.45 units, a population mean using this questionnaire, which was predicted to show a standard deviation of about 4 units. The replacement rate needed was predicted to be 20%.

- Statistical analysis 

The statistical analysis was carried out using the SPSS 23 computer program using a confidence level of 95% and considering statistically significant those comparison results for which the *p-value* obtained is less than 0.05, so that if the *p-value* is less than 0.05 we reject the null hypothesis. Chi-square, Anova and Bonferroni test(s) were used for carry(ing) out the comparison of proportions.

## Results

- Descriptive results 

A total of 250 patients were selected, of those, 199 patients finally completed the study, applying the inclusion and exclusion criteria. Fifty-four patients were regular compliers (27.14%), 53 were erratic compliers (26.63%) and 92 were non-compliers (46.23%). According to the reason for consultation 86 were self-reported (43.22%) while 113 (56.78%) were referred. Patients that had no pain numbered 176 (88.44%) while 23 (11.56%) did feel pain. A number of 166 patients (83.42%) received nonsurgical periodontal treatment while 33 patients (16.58%) also underwent surgery. Half of the participants were male 99 (49.75%) and the other half female 100 (50.25%). The mean age of the sample was 54 years old. There were 66 patients in the 18 to 40 range, which is 33.16% of the sample, 68 in the 41-55 range (34.17%) and 65 patients over 55 (32.66%) (Table 1).

**Table 1:**
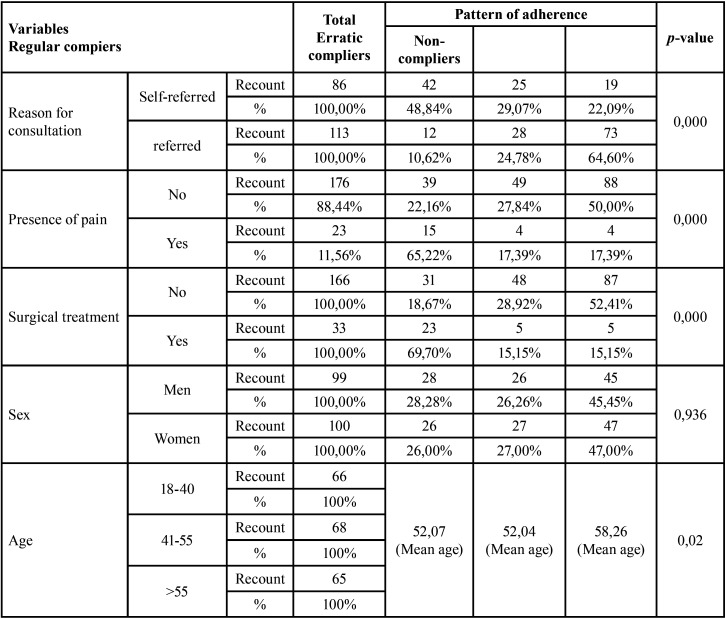
Descriptive results. Relationship between; reason for consultation, pain detection, surgical intervention, sex and age and adherence to SPT. * Using a confidence level of 95% and considering those comparison results statistically significant for which the p-value obtained is less than 0.05. *

-Comparative analysis of the study variables

**Figure 1:**
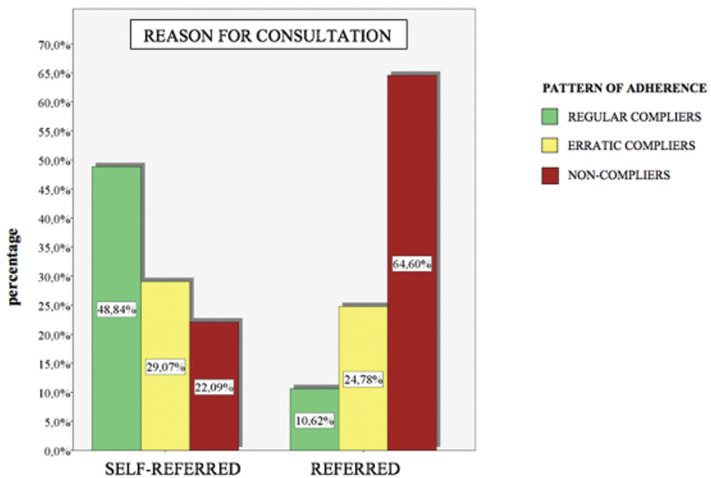
Adherence to SPT depending on the reason for consultation.

**Figure 2:**
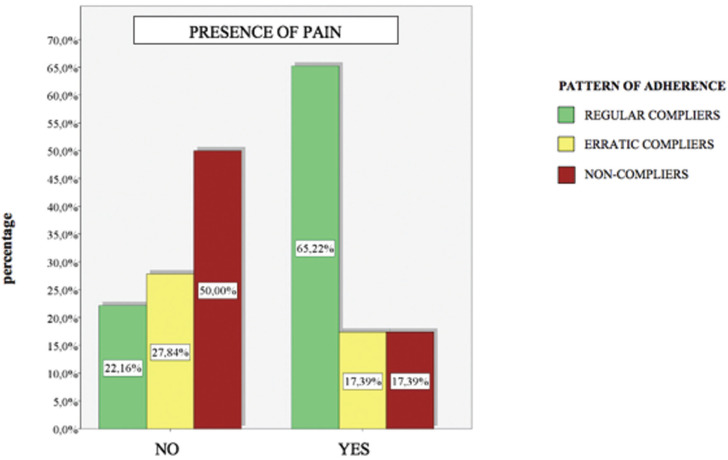
Adherence to SPT and presence of pain.

**Figure 3:**
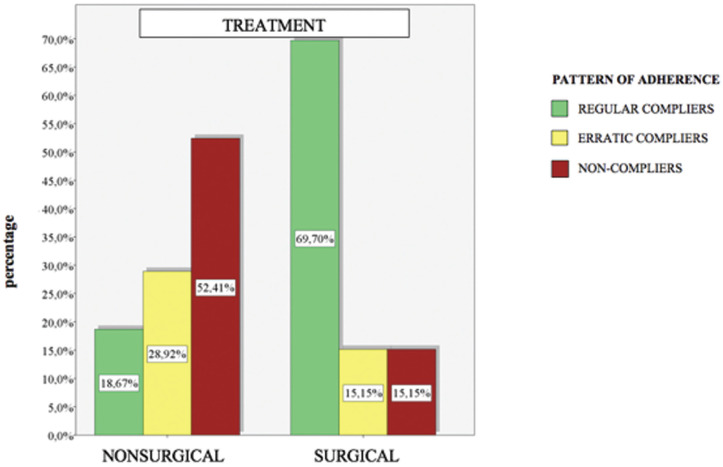
Adherence to SPT and type of treatment received.

**Figure 4:**
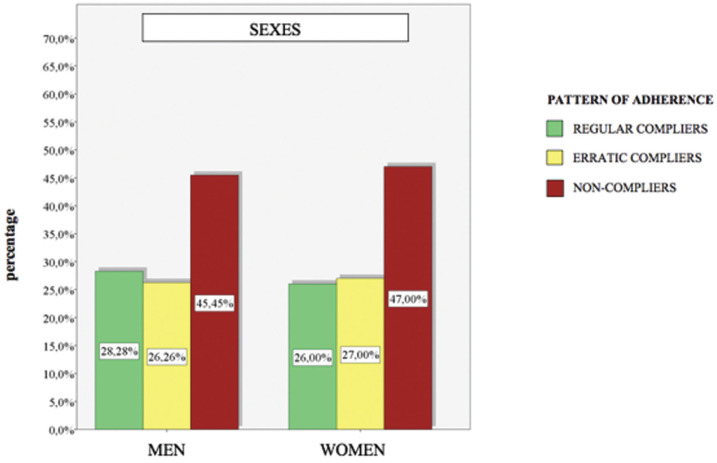
Adherence to SPT between sexes.

- Reason for consultation: The results we obtained in relation to the SPT were that, referring to the reason for consultation in the case of self-referred patients, 48.84% were regular compliers, 29.07% were erratic compliers and 22.09% were non-compliers in attending periodontal treatment support therapy. These self-referred patients (86 patients) 69.77% presented bleeding, 25.58% mobility, 24.42% halitosis, 24.42% gingival recession, 24.42% pain, 18.60% pockets, 18.60% sensitivity and 4.65% bad taste. In the case of referred patients (those who attended for any other reason not related to PD) 10.62% were regular compliers, 24.78% were erratic compliers and 64.60% were non-compliers in attending periodontal treatment support therapy (Fig. 1).

- Presence of pain: In reference to if presence of pain influenced compliance with SPT, 11.56% of patients presented pain. Of those, 65.22% were regular compliers, 17.39% were erratic compliers and 17.39% were non-compliant. Whereas, 88.44% of patients did not detect pain. Of those, 22.46% were regular compliers, 27.84% were erratic compliers and 50% were non-compliers (Fig. 2).

- Surgical/nonsurgical treatment: Depending on whether the patient had undergone basic periodontal treatment or surgical procedures, we discovered that of the patients who underwent surgical treatment, 69.70% were regular compliers, 15.15% were erratic compliers and 15.15% were non-compliant in attending SPT. In the case of patients who received basic periodontal treatment, 18.67% were regular compliers, 28.92% were erratic compliers and 52.41% were non-compliant with SPT (Fig. 3).

- Sociodemographic variables There wasn`t any significant difference between the sexes (*p-value* 0,936). 28,28% of men were regular compliers, and it was 26,00% in the case of women. In the erratic compliers category, we obtained 26,26% of men and it was 27,00% of women. In the non-compliers category, we obtained 45,45% of men and 47,00% of women (Fig. 4).

**Figure 5:**
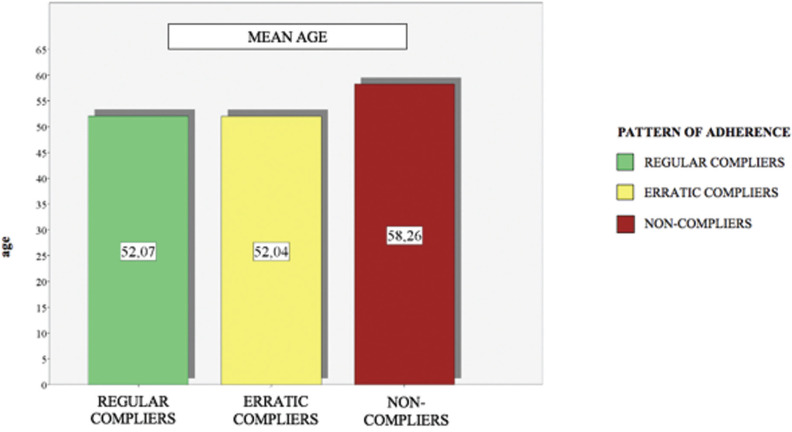
Mean age of regulars, erratic and non-compliant patients.

The mean age for each group of patients with regular adherence was 52.04, the mean age of patients with erratic adherence was 52.04, and the mean age of non- adherent patients was 58.26. Using the Anova test, we found statistical evidence between age and adherence to the SPT. To see what this relationship was, we performed a Bonferroni test (*p*=0.010), in which statistical evidence was obtained to show that older patients were less adherent (Fig. 5).

## Discussion

Given the chronic nature of the disease, long-term SPT is considered a critical pillar of successful treatment. Several studies have shown that in many cases patients give up SPT for different reasons such as age, gender and economic cost (15). In our study we were able to confirm this data since only a percentage of 27.14% showed regular compliance to the SPT, 26.63% showed erratic compliance and 46.23% of patients gave up treatment.

Similar findings were obtained by Delatola C *et al*. 2014 (19) on compliance, where only half of the patients started SPT and only 10.5% continued to attend for 6 years. Renvert *et al*. 2004, found an average of 54% compliance among patients in nine studies in different countries. In many cases, the majority of patients did not attend SPT programmes after the first and second year after active periodontal treatment, moreover, many did not even return for the first appointments (26) Soolari *et al*. 2003 also noted that the overall rate of regular compliance was 3.3%, in contrast with 57.6% erratic compliers and 39.1% non-compliers (27).

Knowing the great impact of this type of disease and the possibility of prevention, what is the reason for these high rates of periodontal disease? According to Echevarría *et al*. 2019, it may be due to three reasons: lack of knowledge, skills or motivation (15).

In regard to the lack of knowledge, in our study approximately half of the patients came referred from another dental specialty such as orthodontics without the capacity for self-diagnosis of PD. This group of patients showed a statistically significant lower adherence to SPT than the self-referred patients. With regard to the referred patients, we observed that only 10.62% were regular adherents, 24.78% were erratic adherents and 64.60% were non-adherents to SPT.

In a clinical study conducted by Varela *et al*. 2019 they obtained similar findings regarding knowledge of the disease, where third of the participants had never heard of periodontitis, and only 1 in 5 respondents could be considered aware of the signs and symptoms of periodontal disease (1). Duque *et al*. 2011 observed a similar proportion of people who were unaware of the existence of PD. We could say that knowledge about PD is still very low and more oral health education is needed (28,29).

Many times patients are not aware of the pathology because in most cases it does not cause pain, which is why it is considered a silent disease. The most common reason for seeking medical and dental treatment is pain and discomfort, therefore, patient motivation towards periodontal treatment and even more SPT can be a challenging aspect (16).

In our study, the percentage of patients who had detected pain was low, 11.56%. All these patients were self-reported, where only 6.15% showed regular compliance. In contrast to our findings, Yeh *et al*. 2011 observed that patients with acute pain symptomatology were more likely to receive periodontal treatment. However, these patients were less likely to complete long- term treatment once the pain had subsided (30).

It has been studied how patient adherence also depends in part on the professional. A systematic review carried out by Newton JT *et al*. 2015 showed the importance of informing the patient, making them understand the benefits following the advice of professionals and thus achieve treatment success. If patients are aware of presenting pathology and have clear goals, monitoring their progress and planning behavioral change is associated with improved outcomes. However, change may not be persistent, for a variety of reasons that disrupts the individual’s habits (31). Fardal *et al*. 2003 noted different cultural and geographical factors in relation to periodontal compliance (32).

Regarding adherence to SPT according to the type of treatment received, we observed that patients who received periodontal surgery were mostly compliant. This may be due to an awareness of the seriousness of the disease, the complex treatment and great economic cost, thus avoiding a second surgery. In the case of patients who have not undergone surgery, just 18.67% were compliant. Cardaropoli *et al*. 2012 also founded noTable differences in adherence among patients who had undergone surgery, suggesting socioeconomic reasons to explain the differences (23). Soolari 2003 obtained similar findings where patients who had received surgery complied better with SPT as opposed to patients who did not undergo surgery (27).

Regarding age, different authors concured that age influences adherence to SPT, showing greater adherence in older patients (33,34). Varela *et al*. 2019 obtained a mean age of 40 to 70 years with greater awareness and compliance with oral hygiene instructions (1). Whereas, other authors didn’t find significant differences between non-compliant and compliant patients in terms of age and gender (32). Bansal *et al*. 2015 observed that the prevalence and severity of PD increased with advancing age because of possible immune impairment and tissue integrity which may increase the severity of the disease, thus may show greater involvement (35). Unlike Agarwal V *et al*. 2010 who advocated the cumulative effect of the untreated PD process over a period of time rather than the aging process (36).

Another sociodemographic variable we studied was sex, where we found no significant differences between men and women (1,19,32,33). However, Famili *et al*. 2010 conducted a study in which they observed that patient compliance was higher in the female sex (37). Bansal *et al*. 2015 advocated that men are less health conscious and have poorer oral hygiene than women (35).

Therefore, when a patient starts periodontal treatment, it will be of great importance to evaluate them and obtain the risk profile, in order to prevent them from giving up SPT and achieve long-term successful treatment.

The present study’s main limitation was that there was just one sampling point. In addition, the sample selection was not random, all the patients were attending periodontics department. Nevertheless, the sample size was adequate and there was just one qualified interviewer so all the patients received the same information and followed the same criteria for classification. Moreover an exhaustive analysis was made, applying Pearson Chi-squared test, Anova, Bonferroni and proportions comparison test.

## Conclusions

As a general conclusion it may be stated that there is an association between the compliance of the SPT and the individual patient-profile; adherence was significantly higher in self-reported patients, patients with acute symptomatology and ones who had underwent surgery. Whereas reported patients from other dental specialties, asymptomatic patients and those who had just received basic periodontal treatment (root scaling and planning) where much less compliant. Talking about gender, we didn’t find conclusive data between men and women. Lastly, we obtained a slight difference between older patients being less compliant than younger ones.

## References

[1] Varela Centelles P, Diz Iglesias P, Estany Gestal A, Blanco Hortas A, Bugarín González R, Seoane Romero JM (2019). Periodontal awareness and what it actually means: a cross-sectional study. Oral Dis.

[2] Behal R, Majid Jan S, Hussain H (2018). Evaluation of the effect of various factors on patient compliance among patients visiting Govt. Dental College and Hospital, Srinagar. IAIM.

[3] Thomson WM, Sheiham A, Spencer AJ (2012). Sociobehavioral aspects of periodontal disease. Periodontol 2000.

[4] Mohd-Dom T, Ayob R, Mohd-Nur A, Abdul-Manaf MR, Ishak N, Abdul-Muttalib K (2014). Cost analysis of periodontitis management in public sector specialist dental clinics. BMC Oral Health.

[5] Graziani F, Karapetsa D, Alonso B, Herrera D (2017). Nonsurgical and surgical treatment of periodontitis: how many options for one disease?. Periodontol 2000.

[6] Krishna R, De Stefano JA (2016). Ultrasonic vs. hand instrumentation in periodontal therapy: clinical outcomes. Periodontol 2000.

[7] Graziani F, Karapetsa D, Mardas N, Leow N, Donos N (2018). Surgical treatment of the residual periodontal pocket. Periodontol 2000.

[8] Jonsson B, Baker SR, Lindberg P, Oscarson N, Ohrn K (2012). Factors influencing oral hygiene behaviour and gingival outcomes 3 and 12 months after initial periodontal treatment: an exploratory test of an extended Theory of Reasoned Action. J Clin Periodontol.

[9] Del Fabbro M, Nevins M, Venturoli D, Weinstein RL, Testori T (2018). Clinically Oriented Patient Maintenance Protocol: A Clinical Consensus of Experts. Int J Periodontics Restorative Dent.

[10] Sonnenschein SK, Kohnen R, Ruetters M, Krisam J, Kim TS (2020). Adherence to long term supportive periodontal therapy in groups with differentperiodontal risk profiles. J Clin Periodontol.

[11] Amerio E, Mainas G, Petrova D, Giner Tarrida L, Nart J, Monje A (2020). Compliance with Supportive Periodontal/Peri-Implant Therapy: A Systematic Review. J Clin Periodontol.

[12] Airila-Mansson S, Bjurshammar N, Yakob M, Söder B (2007). Self-reported oral problems, compared with clinical assessment in an epidemiological study. Int J Dent Hyg.

[13] Manresa C, Sanz-Miralles EC, Twigg J, Bravo M (2018). Supportive periodontal therapy (SPT) for maintaining the dentition in adults treated for periodontitis. Cochrane Database Syst Rev.

[14] Midwood I, Davies M, Newcombe RG, West N (2019). Patients' perception of their oral and periodontal health and its impact: a cross-sectional study in the NHS. Br Dent J.

[15] Echeverría JJ, Echeverría A, Caffesse RG (2019). Adherence to supportive periodontal treatment. Periodontol 2000.

[16] Gokulanathan S, Balan N, Aravind RJ, Thangavelu K (2014). Patient compliance and supportive periodontal therapy: Study among young adults of Namakkal district. J Pharm Bioall.

[17] König J, Plagmann HC, Langenfeld N, Kocher T (2001). Retrospective comparison of clinical variables between compliant and non-compliant patients. J Clin Periodontol.

[18] Costa FO, Cota LOM, Cortelli JR, Cortelli SC, Cyrino RM, Lages EJP (2015). Surgical and Non-Surgical Procedures Associated with Recurrence of Periodontitis in Periodontal Maintenance Therapy: 5-Year Prospective Study. PLoS ONE.

[19] Delatola C, Adonogianaki E, Ioannidou E (2014). Non-surgical and supportive periodontal therapy: Predictors of compliance. J Clin Periodontol.

[20] Sabaté E (2001). WHO Adherence Meeting Report.

[21] Aronson JK (2007). Compliance, concordance, adherence. Br J Clin Pharmacol.

[22] Jn LJ, Armitage GC, Klinge V, Lang NP, Tonetti M, Williams RC (2011). Global oral health inequalities: task group- periodontal diseases. Adv Dent Res.

[23] Cardaropoli D, Gaveglio L (2012). Supportive periodontal therapy and dental implants: an analysis of patients' compliance. Clin Oral Implants Res.

[24] Petersen PE, Ogawa H (2012). The global burden of periodontal disease: towards integration with chronic disease prevention and control. Periodontol 2000.

[25] Arweiler NB, Auschill TM, Sculean A (2017). Patient self-care of periodontal pocket infections. Periodontology 2000.

[26] Renvert S, Persson GR (2004). Supportive periodontal therapy. Periodontol 2000.

[27] Soolari A, Rokn AR (2003). Adherence to periodontal maintenance in Tehran, Iran. A 7- year retrospective study. Quintessence International.

[28] Duque A, Cuartas C, Muñoz C, Salazar C, Sánchez Y (2011). Periodontal knowledge in a sample of employees in Medellín. Revista CES Odontología.

[29] Azodo CC, Ojehanon PI (2012). Does any relationship exist between selfreported gingival bleeding, oral health perception, practices and concerns?. Niger Med J.

[30] Yeh HC, Lai H (2011). Association between patients' chief complaints and their compliance with periodontal therapy. J Clin Periodontol.

[31] Newton JT, Asimakopoulou K (2015). Managing oral hygiene as a riskfactor for periodontal disease: a systematic review of psychological approaches to behaviorchange for improved plaque control in periodontal management. J Clin Periodontol.

[32] Fardal O, Johannessen AC, Linden GJ (2003). Compliance in a Norwegian periodontal practice. Oral Health Prev Dent.

[33] Ojima M, Hanioka T, Shizukuishi S (2001). Survival analysis for degree of compliance with supportive periodontal therapy. J Clin Periodontol.

[34] Ramseier CA, Kobrehel S, Staub P, Sculean A, Lang NP, Salvi GE (2014). Compliance of cigarette smokers with scheduled visits for supportive periodontal therapy. J Clin Periodontol.

[35] Bansal M, Mittal N, Singh TB (2015). Assessment of the prevalence of periodontal diseases and treatment needs: A hospital-based study. J Indian Soc Periodontol.

[36] Agarwal V, Khatri M, Singh G, Gupta G, Marya CM, Kumar V (2010). Prevalence of periodontal diseases in India. J Oral Health Community Dent.

[37] Famili P, Short E (2010). Compliance with periodontal maintenance at the University of Pittsburgh: retrospective analysis of 315 cases. Gen Dent.

